# Femtosecond pulsed laser microscopy: a new tool to assess the in vitro delivered dose of carbon nanotubes in cell culture experiments

**DOI:** 10.1186/s12989-021-00402-5

**Published:** 2021-02-18

**Authors:** Dominique Lison, Saloua Ibouraadaten, Sybille van den Brule, Milica Todea, Adriana Vulpoi, Flaviu Turcu, Christina Ziemann, Otto Creutzenberg, James C. Bonner, Marcel Ameloot, Hannelore Bové

**Affiliations:** 1 Louvain centre for Toxicology and Applied Pharmacology (LTAP), Institut de Recherche Expérimentale et Clinique (IREC), Université catholique de Louvain, Brussels, Belgium; 2 Interdisciplinary Research Institute in Bio- Nano- Sciences, Babes-Bolyai University (BBU), Cluj-Napoca, Romania; 3 Department of Molecular Sciences, Faculty of Medicine, Iuliu Hatieganu University of Medicine and Pharmacy, Cluj-Napoca, Germany; 4 Fraunhofer Institute for Toxicology and Experimental Medicine ITEM, Hannover, Germany; 5 Toxicology Program, Department of Biological Sciences, North Carolina State University, Raleigh, North Carolina USA; 6 Biomedical Research Institute, Hasselt University, Diepenbeek, Belgium

**Keywords:** Particokinetics, Turbidity assay

## Abstract

**Background:**

In vitro models are widely used in nanotoxicology. In these assays, a careful documentation of the fraction of nanomaterials that reaches the cells, i.e. the in vitro delivered dose, is a critical element for the interpretation of the data. The in vitro delivered dose can be measured by quantifying the amount of material in contact with the cells, or can be estimated by applying particokinetic models. For carbon nanotubes (CNTs), the determination of the in vitro delivered dose is not evident because their quantification in biological matrices is difficult, and particokinetic models are not adapted to high aspect ratio materials. Here, we applied a rapid and direct approach, based on femtosecond pulsed laser microscopy (FPLM), to assess the in vitro delivered dose of multi-walled CNTs (MWCNTs).

**Methods and results:**

We incubated mouse lung fibroblasts (MLg) and differentiated human monocytic cells (THP-1) in 96-well plates for 24 h with a set of different MWCNTs. The cytotoxic response to the MWCNTs was evaluated using the WST-1 assay in both cell lines, and the pro-inflammatory response was determined by measuring the release of IL-1β by THP-1 cells. Contrasting cell responses were observed across the MWCNTs. The sedimentation rate of the different MWCNTs was assessed by monitoring turbidity decay with time in cell culture medium. These turbidity measurements revealed some differences among the MWCNT samples which, however, did not parallel the contrasting cell responses. FPLM measurements in cell culture wells revealed that the in vitro delivered MWCNT dose did not parallel sedimentation data, and suggested that cultured cells contributed to set up the delivered dose. The FPLM data allowed, for each MWCNT sample, an adjustment of the measured cytotoxicity and IL-1β responses to the delivered doses. This adjusted in vitro activity led to another toxicity ranking of the MWCNT samples as compared to the unadjusted activities. In macrophages, this adjusted ranking was consistent with existing knowledge on the impact of surface MWCNT functionalization on cytotoxicity, and might better reflect the intrinsic activity of the MWCNT samples.

**Conclusion:**

The present study further highlights the need to estimate the in vitro delivered dose in cell culture experiments with nanomaterials. The FPLM measurement of the in vitro delivered dose of MWCNTs can enrich experimental results, and may refine our understanding of their interactions with cells.

**Supplementary Information:**

The online version contains supplementary material available at 10.1186/s12989-021-00402-5.

## Background

Research efforts to evaluate the health risks of nanomaterials (NM) largely use in vitro approaches and cell culture models. These experiments are relatively easy to perform, but their interpretation is difficult because of the complex distribution of NM in the experimental systems, which can strongly influence the biological outcomes [1]. A careful documentation of the fraction of NM that reaches the cells, i.e. the in vitro delivered dose, is a critical element for the interpretation of in vitro toxicology experiments [2, 3]. This issue is especially important when using submerged cell culture models because the kinetics of the NM suspended in the culture medium (particokinetics) can strongly influence the cellular responses, and can confound the apparent biological activity of the NM. For a proper comparison of the cell responses to various NM, it is essential to integrate differences in particokinetics and, possibly, to adjust the cell responses to the in vitro delivered dose. The determination of the delivered dose is also necessary for the comparison of in vivo and in vitro data, to enable meaningful read-across analyses, and to extrapolate cell data to animal or human exposure conditions for the derivation of exposure limits [3]. Therefore, most toxicology journals now require that cell culture studies with NM provide a documentation of the in vitro delivered dose. This information can be obtained by measuring the dose of NM in contact with the cells, e.g. by quantifying the amount of NM in and/or on the cells [4, 5]. Alternatively, mathematical models are available to estimate particokinetics [6–8].

For CNTs, the documentation of the in vitro delivered dose is challenging because these materials are difficult to quantify chemically in (carbon based) biological matrices. Approaches such as gel electrophoresis [9, 10], programmed thermal analysis [11, 12], Raman spectroscopy [13], and UV-Vis-NIR spectroscopy [14] have been applied to quantify CNTs in biological systems. Indirect approaches based on the measurement of a metallic impurity, e.g. cobalt used as a tracer for CNTs [15], or the use of fluoro- or radio-labeled CNT [16, 17] have also been reported. On the other hand, mathematical models to estimate the particokinetics in cell culture are, up to now, not designed for high aspect ratio NMs, although adaptations have been proposed [18].

In the present study, we applied a rapid and direct approach based on femtosecond pulsed laser microscopy (FPLM) to assess the in vitro delivered dose of multi-walled CNTs (MWCNTs). The principle of FPLM is based on the property of carbonaceous materials, including graphene, fullerenes, single- and multi-walled CNTs to emit white light under femtosecond pulsed illumination [19]. In a previous study, we validated this technology for imaging CNTs in cultured cells [20]. Here, we used two cell lines relevant for the lung response to inhaled MWCNTs, i.e. mouse lung fibroblasts (MLg) and human monocyte-derived macrophages (THP-1) [21, 22], which exhibited contrasting responses to a panel of MWCNTs with varying purity and surface functionalities, all derived from a parent sample, i.e. NC-7000 (Table S1). We found that the in vitro delivered dose of the MWCNTs does not simply result from sedimentation, but is also set up by the cultured cells. Differences in in vitro delivered dose recorded among MWCNT samples might help to explain contrasted cellular responses.

## Results

**Fig. 1:**
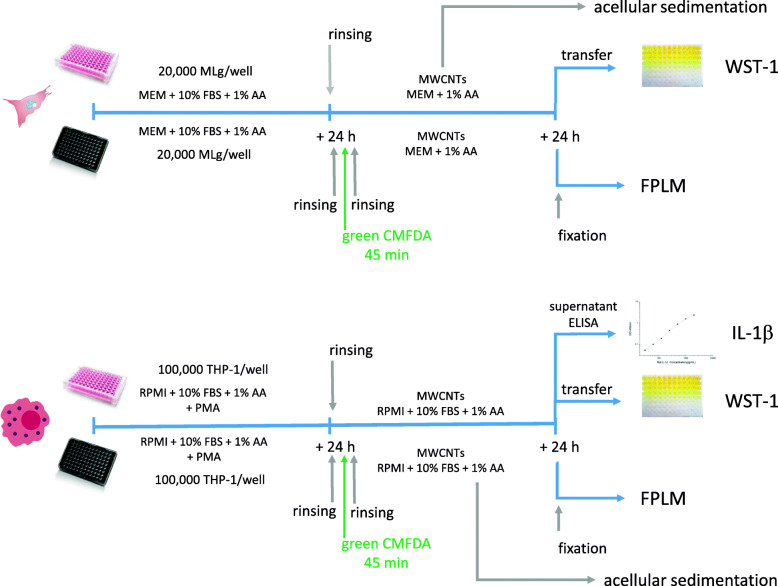
Experimental design

The experimental design (Fig. 1, see Material and methods for details) included two cell types, MLg and THP-1 cells exposed to 2 sets of MWCNTs dispersed in serum-free medium (MEM and RPMI, respectively) at applied doses between 9 and 81 μg/cm^2^ of culture well surface area. This dose range is consistent with the alveolar mass retention of CNTs after a full working lifetime exposure to the current NIOSH recommended exposure level for MWCNTs (1 μg/m^3^) estimated to 12.4–46.5 μg/cm^2^ [23].

The cell responses were recorded with the WST-1 assay for both cell lines, and IL-1β production for THP-1 cells. The sedimentation of the different MWCNTs in the respective culture media was monitored with an acellular turbidity assay. This assay surmises that a reduction in turbidity reflects sedimentation. Cells cultured in parallel in black plates, and exposed to 9 μg/cm^2^ of the same MWCNTs were used for FPLM measurements.

### Mouse lung fibroblasts responses

#### First set of MWCNTs (chemically purified)

**Fig. 2:**
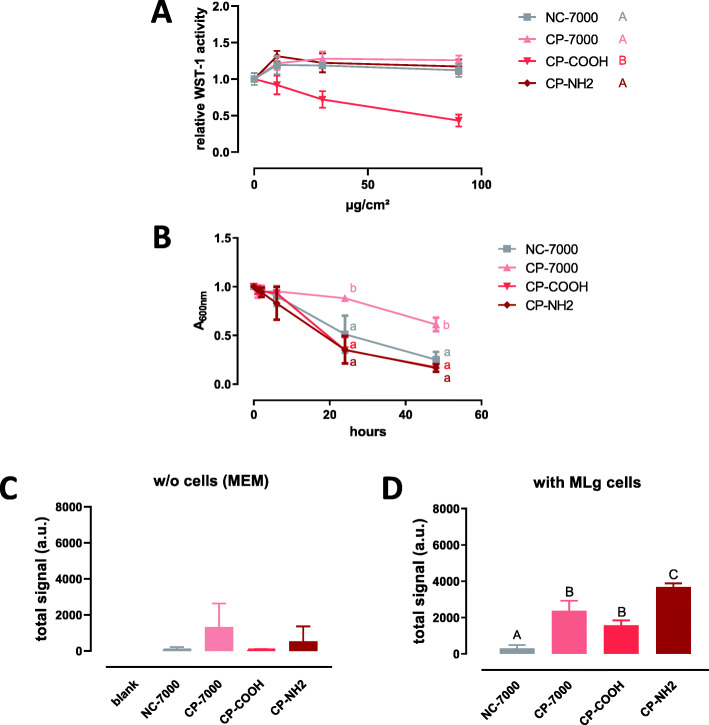
Response of MLg fibroblasts to the first set of MWCNTs. Panel **A** WST-1 assay with MLg cells after 24 h exposure to increasing applied doses of MWCNTs expressed in μg/cm^2^ of culture surface area (mean ± SD, *n* = 4 experimental replicates). Samples with the same letter do not have a statistically different activity [two-ways ANOVA followed by Tukey’s multiple comparison test (mean effect)]. Panel **B** Turbidity kinetics of the different MWCNT samples suspended in serum-free MEM at 50 μg/ml (*n* = 2–6 experimental replicates). Symbols with the same letter are not statistically different (mixed effect model followed by Tukey’s test). Panel **C** Femtosecond pulsed laser signal of deposited MWCNTs after 24 h in the absence (w/o) of cells (a.u., arbitrary unit; mean ± SD; *n* = 3 experimental replicates). Panel **D** Femtosecond pulsed laser signal of deposited MWCNTs after 24 h in the presence of MLg fibroblasts (a.u., arbitrary unit; mean ± SD; *n* = 3 experimental replicates). Columns with the same letter are not statistically different (one-way ANOVA followed by Tukey’s multiple comparison test)

Figure 2a depicts the viability/metabolic activity of MLg as a function of the applied dose. No significant reduction of cell viability/metabolic activity was recorded, except for CP-COOH, which appeared significantly more cytotoxic than the other samples.

We first considered the possibility that differential sedimentation of the MWCNT in the culture wells might contribute to explain the contrasting cell response to CP-COOH. We, therefore, monitored the turbidity decay of the MWCNT suspensions in serum-free MEM (50 μg/ml approximately corresponding to 27 μg/cm^2^ in the WST-1 assay). The sedimentation rate of the CP-COOH sample was not higher compared to all other samples, and the higher WST-1 activity could, therefore, not be explained by higher sedimentation.

We next applied FPLM to directly estimate the deposited dose of MWCNT after 24 h. The FPLM measurements were carried out with a MWCNT suspension (15 μg/ml) corresponding to the lowest applied dose (9 μg/cm^2^) at which differences were recorded in the WST-1 assay. The values recorded for the CP-COOH sample at this dose were statistically significantly different (*p* < 0.0001) compared to all other samples. In the absence of cells, only low signals were recorded for the different samples (Fig. 2c and Figure S1 left). Yet, higher signals were measured in the presence of MLg fibroblasts, with a significantly higher deposition of CP-7000, CP-COOH and CP-NH2 compared to NC-7000 (Fig. 2d and Figure S1 right).

#### Second set of MWCNTs (thermally purified)

**Fig. 3:**
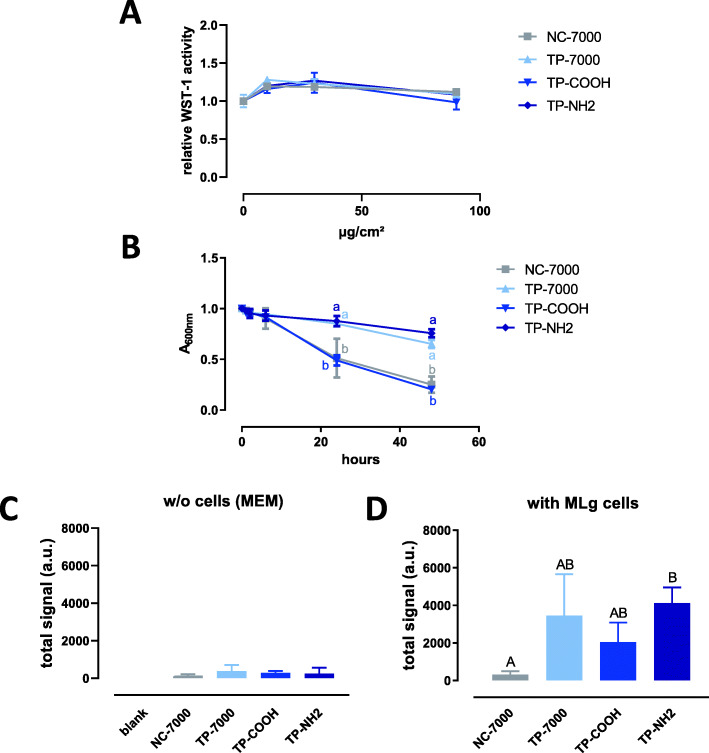
Response of MLg fibroblasts to the second set of MWCNTs. Panel **A** WST-1 assay in MLg cells after 24 h of exposure to increasing applied doses of MWCNTs (mean ± SD; *n* = 4 experimental replicates). Panel **B** Turbidity kinetics of the different MWCNT samples suspended in serum-free MEM at 50 μg/l (*n* = 2–6 experimental replicates). Symbols with the same letter are not statistically different (mixed effect model followed by Tukey’s test). **C** Femtosecond pulsed laser signal of deposited MWCNTs after 24 h in the absence (w/o) of cells (a.u., arbitrary unit; mean ± SD; *n* = 3 experimental replicates). **D** Femtosecond pulsed laser signal of deposited MWCNTs after 24 h in the presence of MLg fibroblasts (a.u., arbitrary unit; mean ± SD; *n* = 3 experimental replicates). Columns with the same letter are not statistically different (one-way ANOVA followed by Tukey’s multiple comparison test)

The MWCNTs did not significantly reduce the viability/metabolic activity of MLg fibroblasts (Fig. 3a). A slower sedimentation was recorded for the TP-7000 and TP-NH2 samples (Fig. 3b). In the absence of cells, a low FPLM signal was detected for all MWCNT samples (Fig. 3c and Figure S2 left). In line with the data obtained with the first set of MWCNTs, a higher signal was measured in the presence of MLg fibroblasts. Significantly more TP-NH2 was deposited compared to the NC-7000 sample (Fig. 3d).

### THP-1 macrophage responses

The viability/metabolic activity of THP-1 macrophages and their production of the pro-inflammatory cytokine IL-1β were assessed after 24 h exposure to the same sets of MWCNTs than in experiments with MLg cells.

#### First set of MWCNTs (chemically purified)

**Fig. 4:**
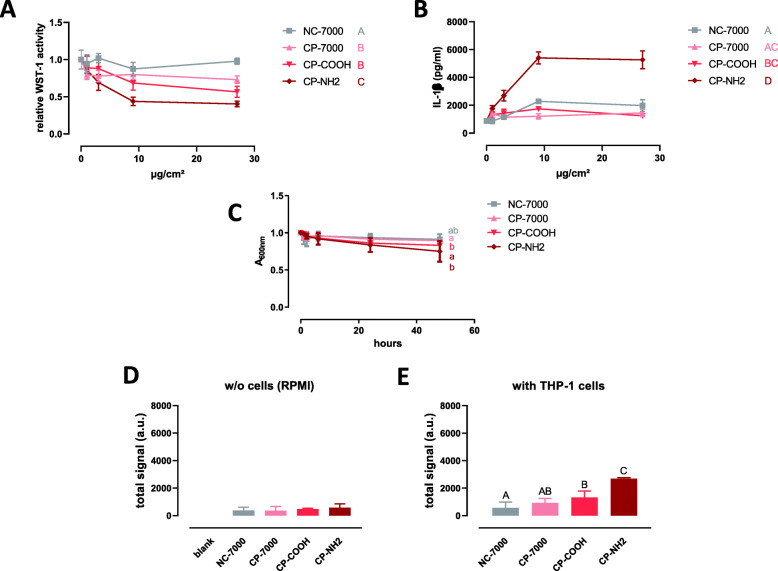
Response of THP-1 macrophages to the first set of MWCNTs. Panel **A** WST-1 assay in THP-1 cells after 24 h of exposure to increasing applied doses of MWCNTs (mean ± SD; *n* = 4 experimental replicates). Samples with the same letter do not have a statistically different activity [two-ways ANOVA followed by Tukey’s multiple comparison test (mean effect)]. Panel **B** IL-1β secretion by THP-1 cells after 24 h of exposure to increasing applied doses of chemically purified MWCNTs (mean ± SD; *n* = 4 experimental replicates). Samples with the same letter do not have a statistically different activity [two-ways ANOVA followed by Tukey’s multiple comparison test (mean effect]). Panel **C** Turbidity kinetics of the different MWCNT samples suspended in serum-free RPMI at 50 μg/l (*n* = 2–6 experimental replicates). Symbols with the same letter are not statistically different (mixed effect model followed by Tukey’s test). Panel **D** Femtosecond pulsed laser signal of deposited MWCNTs after 24 h in the absence (w/o) of cells (a.u., arbitrary unit; mean ± SD; *n* = 3 experimental replicates). Panel **E** Femtosecond pulsed laser signal of deposited MWCNTs after 24 h in the presence of THP-1 cells (a.u., arbitrary unit; mean ± SD; *n* = 3 experimental replicates). Columns with the same letter are not statistically different (one-way ANOVA followed by Tukey’s multiple comparison test)

Samples CP-7000, CP-COOH and CP-NH2 were significantly more cytotoxic to THP-1 macrophages than NC-7000, and CP-NH2 exhibited the highest cytototoxic activity (Fig. 4a). In the IL-1β assay, CP-NH2 was also the most active sample compared to NC-7000, CP-7000 and CP-COOH (Fig. 4b). In the turbidity assay, the MWCNT suspensions appeared more stable in serum-free RPMI than in serum-free MEM, with slight differences among the samples (Fig. 4c). In the absence of cells, low FPLM signals were recorded for all the samples (Fig. 4d and Figure S3 left). Again, higher deposited doses of MWCNTs were recorded in the presence of cells, with significantly more deposited CP-NH2 compared to other samples (Fig. 4d).’

#### Second set of MWCNTs (thermally purified)

**Fig. 5:**
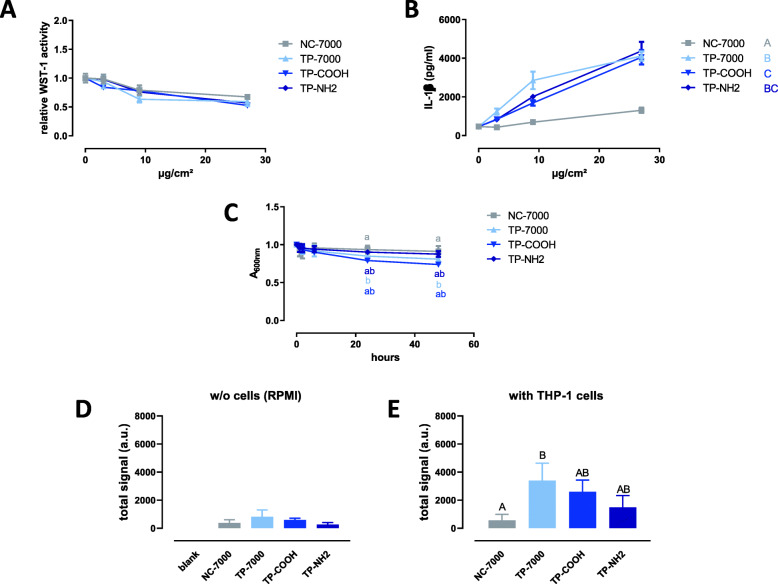
Response of THP-1 macrophages to the second set of MWCNTs. **A** WST-1 assay in THP-1 cells after 24 h of exposure to increasing applied doses of MWCNTs (mean ± SD; *n* = 4 experimental replicates). Samples with the same letter do not have a statistically different activity [two-ways ANOVA followed by Tukey’s multiple comparison test (mean effect)]. **B** IL-1β secretion by THP-1 cells after 24 h of exposure to increasing applied doses of MWCNTs (mean ± SD; *n* = 4 experimental replicates). Samples with the same letter do not have a statistically different activity [two-ways ANOVA followed by Tukey’s multiple comparison test (mean effect)]. **C** Turbidity kinetics of the different MWCNT samples suspended in RPMI at 50 μg/ml (*n* = 2–6 experimental replicates). Symbols with the same letter are not statistically different (mixed effect model followed by Tukey’s test). **D** Femtosecond pulsed laser signal of deposited MWCNTs after 24 h in the absence (w/o) of cells (a.u., arbitrary unit; mean ± SD; *n* = 3 experimental replicates). **E** Femtosecond pulsed laser signal of deposited MWCNTs after 24 h in presence of THP-1 cells (a.u., arbitrary unit; mean ± SD; *n* = 3 experimental replicates). Columns with the same letter are not statistically different (one-way ANOVA followed by Tukey’s multiple comparison test)

The TP-7000, TP-COOH and TP-NH2 samples slightly affected the viability/metabolic activity of THP-1 macrophages, with no statistical difference among the samples (Fig. 5a). These samples induced a higher production of IL-1β compared to NC-7000 (Fig. 5b). At the applied dose of 9 μg/cm^2^, TP-7000 was the most active sample to produce IL-1β (*p* < 0.01 and *p* < 0.05 compared to TP-COOH and TP-NH2, respectively; Tukey’s multiple comparison test). The slope of the turbidity decay in serum-free RPMI was similar for all MWCNT samples (Fig. 5c). In the absence of cells, low FPLM signals were recorded for all MWCNT samples (Fig. 5d). Higher FPLM signals were recorded in the presence of THP-1 macrophages, with a significantly higher deposition of TP-7000 compared to other samples (Fig. 5e).

## Discussion

The focus of the present study was to use FPLM for the estimation of the in vitro delivered dose of different MWCNTs in submerged cell cultures. We observed contrasting cell responses to the MWCNT samples, both in MLg fibroblasts and in THP-1 macrophages. When considering the nominal applied dose (μg/cm^2^), the CP-COOH sample appeared the most cytotoxic in MLg fibroblasts (Fig. 2a), whereas CP-NH2 was the most cytotoxic sample and induced the highest release of IL-1β in THP-1 macrophages (Fig. 4a and b). The TP-7000, TP-COOH and TP-NH2 samples induced a higher release of IL-1β from THP-1 macrophages compared to the parent sample NC-7000 (Fig. 5b). These contrasting responses might reflect either a higher intrinsic biological activity of these MWCNT samples and/or a higher delivery to the cells. We approached the in vitro delivered dose by estimating the sedimentation kinetics of the various MWCNT suspended in the culture media, and by estimating the amount of deposited MWCNT using FPLM. We estimated the sedimentation kinetics by measuring the decay in turbidity of MWCNT suspensions in serum-free culture media. MWCNT suspensions in RPMI appeared more stable than in MEM, possibly reflecting the higher amino acid content of RPMI. Indeed, amino acids can enhance the dispersion of CNTs by increasing electrostatic repulsive interactions between nanotubes [24]. However, the MWCNT samples that sedimented the fastest were not the most active MWCNTs in the cellular assays. Thus, these turbidity measurements did not appear contributive to explain the contrasting cellular responses to the MWCNT samples. Next, we estimated the delivered MWCNT dose by FPLM. This imaging technology allows the label-free detection of carbon-based materials in biological samples. FPLM does not require prior processing of the samples, such as mineralization for NIR spectroscopy [14]. As the emission of white light under femtosecond pulsed laser illumination results from the heterogeneous and absorptive nature of the carbon particles, MWCNT impurities and/or surface functional moieties do not have a substantial impact on the FPLM signal (Figure S5A). Thus, FPLM offers a clear advantage compared to Raman spectroscopy, where the purity and the functionalization of CNTs can influence the signal. Another advantage of FPLM is that different observation wavelengths can be used, which is especially useful when a spectral array detector is not available on the microscope, or when the combination with different fluorescent fluorophores is desired. The possibility to use different wavelengths also minimizes the impact of the background from biological samples, which often interferes when using Raman spectroscopy. A limitation of the FPLM method is, however, that, at high doses, particles located in different positions along the optical axis of the microscope can be superimposed, possibly leading to partial masking, and underestimation of the signal. This can be addressed by capturing images at different positions along the optical axis throughout the MWCNT layer. In the present study, we used a low MWCNT dose (9 μg/cm^2^) to minimize superimposition, and the plane of focus was at the middle of the cell layer. We expressed the FPLM signal in arbitrary units to compare the deposited dose across different samples. A calibration of the FPLM measurements to mass or nanotube number dose would be required to increase the transferability of these measurements, e.g. to extrapolate the MWCNT to in vivo models or for risk assessment purposes. The signals measured in wells with cells were markedly higher than in wells without cells, indicating a strong attachment of the MWCNT to the cell layer. Several dose metrics can be used to characterize the FPLM images, including total particle signal, total particle area, cell-associated particle signal, cell-associated particle area, etc. Here, we report the total signal of deposited MWCNTs because we do not have an a priori hypothesis on the scenario of interaction between MWCNTs and cells. MWCNTs can act in the cells [25, 26] or on the cell surface, e.g. by interacting with surface receptors [10, 27, 28]. MWCNTs next to the cells, e.g. bound to extracellular products, might also be important to consider in toxicology assays. As part of the FPLM signals (between approximately 40–87%) did not co-localize with the cell staining, the total signal might also include MWCNTs attached to extracellular products released by the cells. The presence of secreted proteins, cell products or debris can accelerate the deposition of MWCNT in cell culture [14]. Importantly, this cell-dependent FPLM signal differed across the MWCNT samples, and these differences were not consistent with turbidity data. The influence of the cell layer on the deposited dose, therefore, suggests that sedimentation, as assess by acellular turbidity measurements, cannot capture all the events that contribute to the MWCNT dose delivered to submerged cells. In fibroblast cultures, FPLM measurements indicated that the in vitro delivered dose differed significantly across MWCNTs. These results suggest, in particular, that the strong reduction of viability/metabolic activity observed in fibroblasts exposed to the CP-COOH sample (Fig. 2a) reflects an intrinsically higher cytotoxic activity of this sample because the deposited dose for this sample was not higher than that of the other samples. Interestingly, CP-COOH was not the most active sample in macrophages. In macrophages, CP-NH2 exhibited the highest cytotoxic and pro-inflammatory potential, which might be explained by the higher delivered dose of the CP-NH2 sample as compared to the other samples (Fig. 4a and b, respectively).

**Fig. 6:**
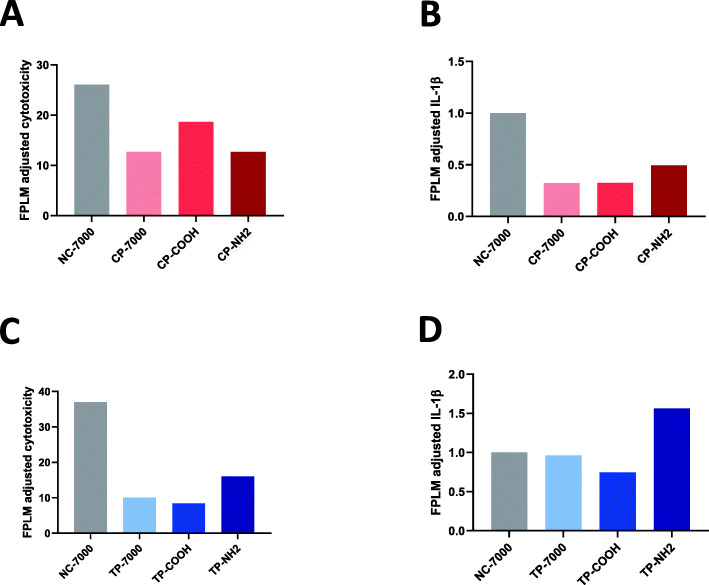
Macrophage responses to the first (**A**, **B**) and second set of MWCNTs **(C**, **D**) tentatively adjusted to the in vitro delivered dose as determined by FPLM. For cytotoxicity data (panels **A** and **C**), the relative (remaining) WST1 activities recorded at 9 μg/ml in THP-1 cells (Figs. 4a and 5s, *n* = 4) was transformed to reflect the net cytotoxic activity (i.e., 1 - relative WST-1 activity). For each CNT sample, the mean of these values was calculated. This mean was then divided by the mean FPLM signal recorded in THP-1 cells after exposure to the same applied dose (9 μg/ml) (Figs. 4e and 5e, *n* = 3). The same procedure was applied for IL-1β (panels B and D), i.e., for each CNT sample, the mean of the IL-1β concentrations recorded at 9 μg/ml was calculated (Figs. 4b and 5b, *n* = 4) and then divided by the mean FPLM signal recorded in THP-1 cells after exposure to 9 μg/ml (Figs. 4e and 5e, *n* = 3)

The responses recorded in THP-1 macrophages, which express scavengers receptors that bind MWCNT and contribute to their cellular uptake [28], highlight the importance of integrating the in vitro delivered dose for the interpretation of the experimental results. In the absence of FPLM data, the conclusion would be that the cytotoxic activity of MWCNTs increases from NC-7000 to CP-NH2, and that CP-NH2 possesses an enhanced capacity to release IL-1β. When adjusting the results to the in vitro delivered dose, as estimated by FPLM (Fig. 6a), the conclusion appears very different. For the second set of samples (thermally purified), the apparent conclusion in the absence of FPLM data would be that the cytotoxic activity did not differ among the samples, while samples TP-7000, TP-COOH and TP-NH2 enhanced the capacity of THP-1 cells to release IL-1β compared to NC-7000. The interpretation appears very different after adjustment to the in vitro delivered dose (Fig. 6b). After adjustment, the cytotoxic activity of MWCNTs TP-7000 and TP-NH2 appears reduced compared to NC-7000. The latter conclusion is consistent with existing knowledge that thermal treatment (TP-7000, TP-COOH, TP-NH2; Table S1) generally reduces the toxicity of MWCNTs by depleting metallic contaminants and annealing structural defects [29].

Thus, the present results further highlight the need to document the delivered dose in in vitro investigations with NMs, especially with MWCNTs [3, 30]. The integration of the in vitro delivered dose can enrich the experimental results, and may refine our understanding of the interactions of MWCNTs with cells. However, the experimental results should not necessarily, or systematically be adjusted to the in vitro delivered dose. Indeed, for a sample that is highly taken up by the cells and that consequently induces a strong cytotoxic response (possibly CP-NH2 in the present study), this uptake property should be considered as an integral component of the cytotoxic activity of the sample, and an adjustment for the cellular dose might not be justified. It also remains to be explored at which time point the in vitro delivered dose should be determined. It is indeed not immediately evident that the cellular responses and delivered dose should be determined at the same time point. The delivered dose should rather be measured at an earlier time point than the cell response. Appropriate time points may also vary according to the NMs and cell type considered. Thus, the in vitro delivered dose remains a complex issue in nanotoxicology. Several questions remain open, and require a nuanced and critical assessment.

## Conclusion

Assessing the delivered dose of carbon nanomaterials in cell culture is not evident in biological matrices, and this work demonstrates that an estimate of the delivered dose can be obtained by using FPLM. FPLM data can help adjusting the effects according to the delivered dose, opening a new horizon for the refinement of in vitro toxicology of carbon materials.

## Materials and methods

### MWCNT samples and characterization

A panel of custom-synthesized, purified and functionalized multi-walled CNTs was obtained from Nanocyl (Sambreville, Belgium). The physicochemical characteristics of the 7 samples (the parent sample NC-7000, the chemically-purified (CP) CP-7000, CP-COOH and CP-NH2, and thermally purified (TP) TP-7000, TP-COOH and TP-NH2) were already reported in detail by Taylor-Just et al. [31] and are summarized in Table S1.

### Cellular models

Normal mouse lung fibroblasts (MLg, ATCC catalog n°CCL-206) were cultured in minimal essential medium (MEM, Invitrogen) supplemented with 10% fetal bovine serum (FBS, Invitrogen) and 1% of antibiotic-antimycotic (AA, Invitrogen) at 37 °C with 5% CO_2_. The cells were distributed in 96-well culture plates (Cellstar, Ref 655180, Greiner) at a density of 20,000 cells per well in complete medium (10% FBS, 1% AA). THP-1 monocytes (ATCC catalog n°TIB-202) were cultured in RPMI-1640 (Invitrogen) supplemented with 10% FBS, 1% AA, 1 mM sodium pyruvate (Invitrogen) and 10 mM HEPES (Invitrogen) at 37 °C with 5% CO_2_. The cells were distributed in a 96-well culture plate at a density of 100,000 cells per well in complete medium (10% FBS, 1% AA, 1 mM sodium pyruvate, 10 mM HEPES) and differentiated into macrophages by addition of 100 nM phorbol 12-myristate 13-acetate (PMA, Sigma Aldrich). The cells were cultured at 37 °C for 24 h before MWCNT exposure.

### MWCNT suspensions and cell exposure

Stable MWCNT suspensions were prepared immediately before cell exposure according to a procedure described previously [21]. Briefly, MWCNTs were weighed in sterile glass vials, heated at 200 °C for 2 h to inactivate any endotoxin contamination, and suspended at a concentration of 2.55 mg/ml sterile nanopure water (Barnstead NANOPure, Thermo-Fischer) containing 1.4 mg bovine serum albumin/ml (0.14% BSA, Sigma Aldrich). These suspensions were sonicated (VC750 Ultrasonic processor, Sonics & Materials) on ice for 16 min at 40% of maximal power with a 13 mm probe. Next, the suspensions were serially diluted with 0.14% BSA to achieve the finally applied doses of 9 to 81 μg MWCNT/cm^2^ (well surface area) after 10-fold dilution with the culture medium. The cells were incubated for 24 h with 180 μl of serum-free medium (MEM or RPMI + sodium pyruvate + HEPES) + 20 μl of the respective MWCNT dilution in 0.14% BSA.

### WST-1 assay

Cell viability/metabolic activity was evaluated by the WST-1 assay (Roche Diagnostics). After MWCNT exposure, the cells were rinsed with their respective serum-free medium, and incubated at 37 °C with 200 μl of WST-1 diluted in serum-free medium (1:10 for MLg; 1:20 for THP-1). Absorbance was recorded at 480 nm (corrected to 680 nm) with a spectrophotometer (Infinite F200, Tecan). When the absorbance of the control cells reached at least 0.5, the plates were centrifuged and 150 μl of supernatant was transferred to a new 96-well plate and the absorbance was measured. This procedure eliminates the interference of MWCNTs with absorbance. The results are expressed as a relative activity compared to control cells.

### IL-1β assay

THP-1 supernatants were collected after centrifugation of the culture plates, and human IL-1β was quantified using an ELISA kit for IL-1β/IL-1F2 according to the manufacturer’s instructions (detection limit: 3.91 pg/ml, Ref DY201, R&D Systems).

### Turbidity assays

Stock suspensions of MWCNT (2.55 mg/ml) were prepared as described above. These suspensions were diluted to 500 μg/ml with 0.14% BSA. The suspensions were then diluted to 50 μg/ml in serum-free medium (MEM or RPMI + sodium pyruvate + HEPES) in disposable spectrophotometric cuvettes, and were stored vertically on a bench at room temperature. Absorbance was measured at 600 nm with a spectrophotometer (Ultrospec 3000, Pharmacia Biotech) after 0, 1, 2, 6, 24 and 48 h.

### Preparation of the cells for FPLM

Stock suspensions of MWCNTs (2.55 mg/ml) were prepared as described above, and diluted with serum-free culture medium to reach a final concentration of 15 μg/ml in 0.14% BSA. The cells (MLg or THP-1) were cultured in black 96-well plates (Screenstar, Ref 655,866, Greiner) as described above. After 24 h to enable cell adhesion, the cells were stained for 45 min with 5 μM of the vital stain Cell Tracker Green CMFDA (Invitrogen). The cells were then washed, and exposed to a concentration of 15 μg/ml of the respective MWCNTs (9 μg/cm^2^ in 200 μl exposure medium). Wells without cells were treated similarly in parallel. After 24 h, the supernatants were poured off, the wells were fixed with 1.25% paraformaldehyde for 20 min, and covered with 50 μl Immu-Mount (Thermo Fischer Scientific) for FPLM imaging.

### Femtosecond pulsed laser microscopy

The delivered MWCNT dose was imaged according to a method reported previously for detecting CNTs in a biocompatible and label-free manner using femtosecond pulsed laser microscopy [19]. All imaging experiments were performed on a Zeiss LSM 880 (Carl Zeiss, Jena, Germany) inverted confocal laser-scanning microscope using a Zeiss EC Plan-Neofluar 20x/0.50 M27 objective.

The emission fingerprints of the MWCNTs were collected from wells without cells under femtosecond pulsed laser excitation (approximately 10 mW laser power at the samples, 810 nm, 130 fs, 80 MHz, MaiTai DeepSee, Spectra Physics, USA). The gain and/or laser power were adapted according to the intensity of the emission signal to avoid detector saturation and to ensure sufficient emission light detection. After spectral separation of the excitation and emission light, the signals ranging between 405 and 690 nm using a QUASAR thirthy-two channel GaASP spectral detector of a LSM 880 confocal microscope. For each MWCNT, 3 technical replicates of 3 experimental replicates, and thus a total of 9 spectra, were analyzed. These measurements indicated that the emission fingerprints of the different MWCNT all showed white-light generation over the whole visible spectrum (Figure S5A), and the mean intensity values of the spectrum recorded between 400 and 410 nm, i.e. the detection range used in this study (see below), showing no significant differences between the pristine and chemically or thermally purified MWCNTs (Figure S5B).

For imaging the deposited MWCNTs at the bottom of the culture wells, a 488-nm Ar-ion laser (approximately 4 μW radiant power at the sample, S170C microscope slide power sensor, Thorlabs, Munich, Germany) was used as excitation source, and the emission light was filtered between 500 and 550 nm. MWCNTs were visualized by femtosecond pulsed laser excitation (approximately 5 mW laser power at the samples, 810 nm, 130 fs, 80 MHz, MaiTai DeepSee) and the emitted signal was filtered using a 400–410 nm band-pass filter in non-descanned mode. The resulting 512 × 512 images with a pixel size of 0.92 μm were recorded at a pixel dwell time of 8.2 μs. Images were processed with the Fiji program (ImageJ 2.0.0-rc-69, open source software, http://fiji.sc/Fiji). A threshold was set to the estimated background value on the image, and the total MWCNT signal, i.e. the number of counts detected in the image, was determined. For each condition, 3 experimental replicates (i.e. 3 different wells per condition) with 3 technical replicates (i.e. 3 different random regions of interest), and thus a total of 9 images were analyzed. For all images, the plane of focus was at the middle of the cell layer.

### Statistics

Data were analyzed with the GraphPad Prism software (version 8.3.1, GraphPad Software, San Diego, CA). Values represent the arithmetic mean ± standard deviation of 3 experimental replicates (3 wells). Differences between MWCNTs were assessed by conducting a 1-way, 2-way ANOVA or mixed effect model, as appropriate, followed by a Tukey’s multiple comparison test. A *p*-value < 0.05 was considered statistically significant.

## Supplementary Information

**Additional file 1: Table S1.** Summary of the MWCNT characteristics see Taylor-Just for details. **Figure S1.** FPLM detection of chemically purified MWCNT in absence or presence of fibroblasts. Femtosecond pulsed laser microscopy imaging of chemically purified MWCNT (red) deposited after 24 h in the absence (w/o, left) and presence (right) of MLg fibroblasts (green). Scale bars: 50 μm. **Figure S2.** FPLM detection of thermally purified MWCNT in absence or presence of fibroblasts. Femtosecond pulsed laser microscopy imaging of thermally purified MWCNT (red) deposited after 24 h in the absence (w/o, left) and presence (right) of MLg fibroblasts (green). Scale bars: 50 μm. **Figure S3.** FPLM detection of chemically purified MWCNT in absence or presence of macrophages. Femtosecond pulsed laser microscopy imaging of chemically purified MWCNT (red) deposited after 24 h in the absence (w/o, left) and presence (right) of THP-1 macrophages (green). Scale bars: 50 μm. **Figure S4.** FPLM detection of thermally purified MWCNT in absence or presence of macrophages. Femtosecond pulsed laser microscopy imaging of thermally purified MWCNT (red) deposited after 24 h in the absence (w/o, left) and presence (right) of THP-1 macrophages (green). **Figure S5.** Validation of the emission signal of the different MWCNTs. (A) Emission fingerprint of the MWCNTs under femtosecond pulsed laser illumination (excitation 810 nm, 80 MHz, about 10 mW laser power on the sample) confirming the white-light generation of each MWCNT. Intensity normalized to the highest value of each spectrum. For each MWCNT, the spectrum represents the mean of 9 spectra (three technical replicates of three experimental replicates). Spectra are recorded from the wells without cells. (B) Comparison of the mean ± standard deviation intensity values of the spectrum recorded between 400 and 410 nm. No significant difference among the MWCNTs (one-way ANOVA followed by Tukey’s multiple comparison test).

## Data Availability

The samples and datasets used and analyzed in the current study are available from the corresponding author on reasonable request.

## Abbreviations

AA: Antibiotic-antimycotic
CNT: Carbon nanotube
FBS: Fetal bovine serum
FPLM: Femtosecond-pulsed laser microscopy
MLg: Mouse lung fibroblasts
MEM: Modified Eagle’s medium
MWCNT: Multiwalled carbon nanotube
NM: Nanomaterial
RPMI: Rosewell Park Memorial Institute medium
