# Supplementary material for: Femtosecond pulsed laser microscopy: a new tool to assess the in vitro delivered dose of carbon nanotubes in cell culture experiments
Source: Part Fibre Toxicol. 2021 Feb 18;18:9. doi: 10.1186/s12989-021-00402-5 (PMC7890618; doi:10.1186/s12989-021-00402-5)
Supplement: Supplementary file 1 — Additional file 1: Table S1. Summary of the MWCNT characteristics see Taylor-Just for details. Figure S1. FPLM detection of chemically purified MWCNT in absence or presence of fibroblasts. Femtosecond pulsed laser microscopy imaging of chemically purified MWCNT (red) deposited after 24 h in the absence (w/o, left) and presence (right) of MLg fibroblasts (green). Scale bars: 50 μm. Figure S2. FPLM detection of thermally purified MWCNT in absence or presence of fibroblasts. Femtosecond pulsed laser microscopy imaging of thermally purified MWCNT (red) deposited after 24 h in the absence (w/o, left) and presence (right) of MLg fibroblasts (green). Scale bars: 50 μm. Figure S3. FPLM detection of chemically purified MWCNT in absence or presence of macrophages. Femtosecond pulsed laser microscopy imaging of chemically purified MWCNT (red) deposited after 24 h in the absence (w/o, left) and presence (right) of THP-1 macrophages (green). Scale bars: 50 μm. Figure S4. FPLM detection of thermally purified MWCNT in absence or presence of macrophages. Femtosecond pulsed laser microscopy imaging of thermally purified MWCNT (red) deposited after 24 h in the absence (w/o, left) and presence (right) of THP-1 macrophages (green). Figure S5. Validation of the emission signal of the different MWCNTs. (A) Emission fingerprint of the MWCNTs under femtosecond pulsed laser illumination (excitation 810 nm, 80 MHz, about 10 mW laser power on the sample) confirming the white-light generation of each MWCNT. Intensity normalized to the highest value of each spectrum. For each MWCNT, the spectrum represents the mean of 9 spectra (three technical replicates of three experimental replicates). Spectra are recorded from the wells without cells. (B) Comparison of the mean ± standard deviation intensity values of the spectrum recorded between 400 and 410 nm. No significant difference among the MWCNTs (one-way ANOVA followed by Tukey’s multiple comparison test). [file 12989_2021_402_MOESM1_ESM.docx]

**Femtosecond pulsed laser microscopy: a new tool to assess the delivered dose of carbon nanotubes in cell cultures**

Dominique Lison et al.

**Supplementary data**

**Table S1 : Summary of the MWCNT characteristics see Taylor-Just for details^[[1]](#footnote-1)^**

| **MWCNT code** | **Purification** | **Purity** | **Surface function** |
| --- | --- | --- | --- |
| NC-7000 | - |  | - |
| CP-7000 | chemical | + | - |
| CP-COOH | chemical | + | carboxyl |
| CP-NH2 | chemical | + | amine |
| TP-7000 | thermal | ++ | - |
| TP-COOH | thermal | ++ | carboxyl |
| TP-NH2 | thermal | ++ | amine |

***
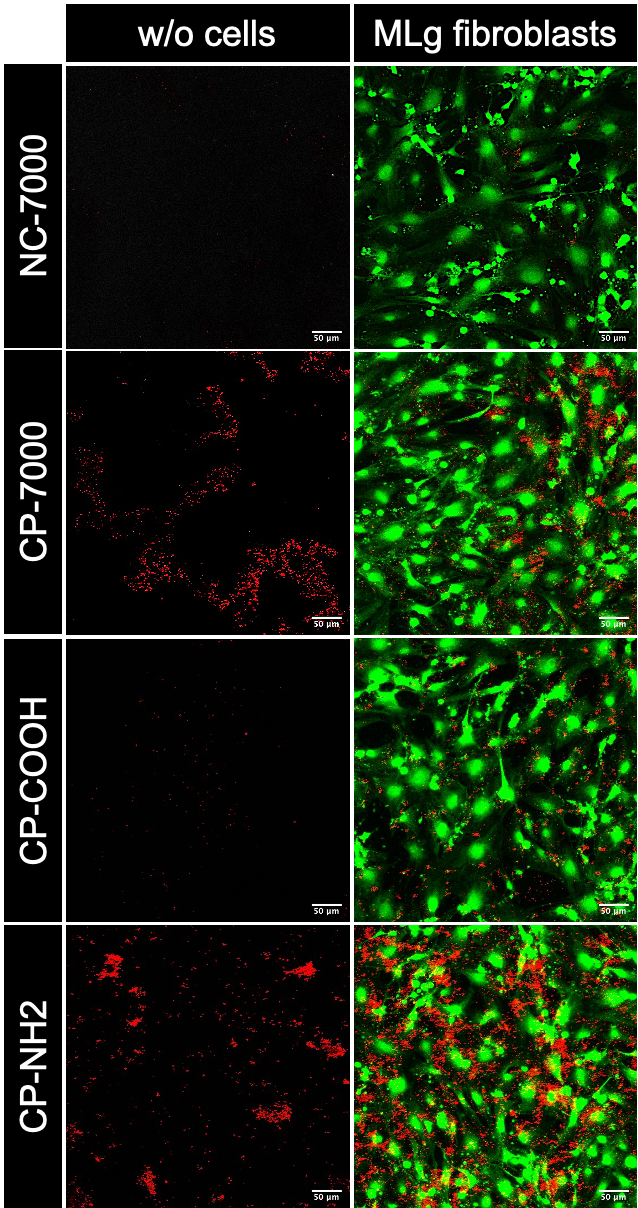
***

***Figure S1: FPLM detection of chemically purified MWCNT in absence or presence of fibroblasts.*** Femtosecond pulsed laser microscopy imaging of chemically purified MWCNT (red) deposited after 24 h in the absence (w/o, left) and presence (right) of MLg fibroblasts (green). Scale bars: 50 μm.

*
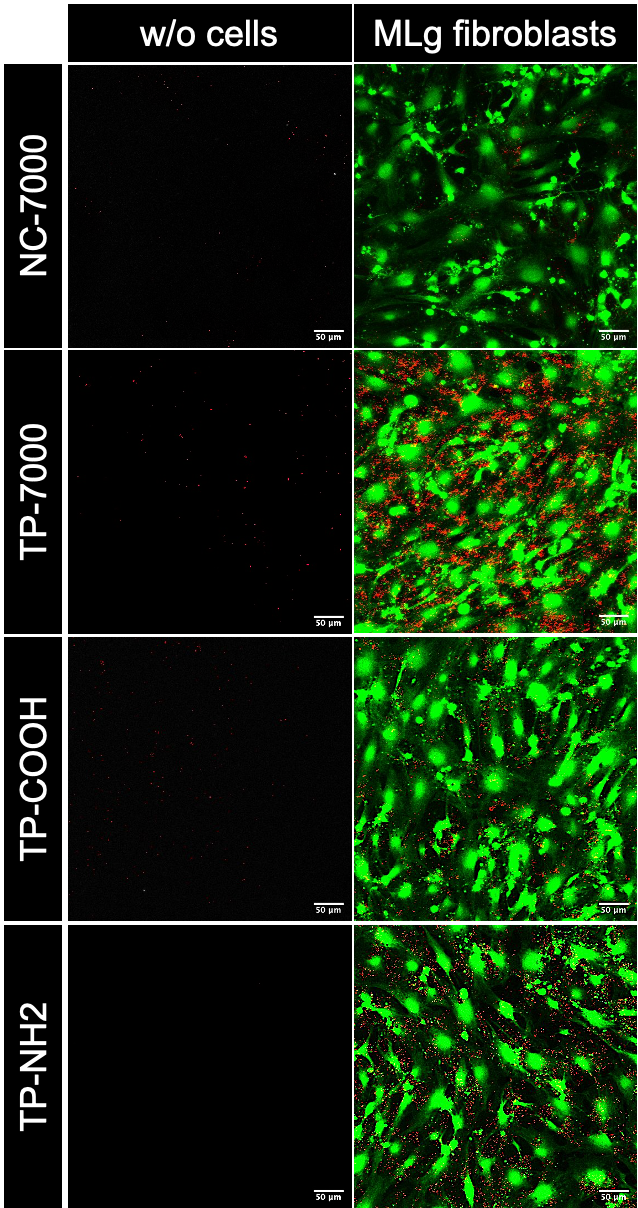
*

***Figure S2: FPLM detection of thermally purified MWCNT in absence or presence of fibroblasts.*** Femtosecond pulsed laser microscopy imaging of thermally purified MWCNT (red) deposited after 24 h in the absence (w/o, left) and presence (right) of MLg fibroblasts (green). Scale bars: 50 μm.

***
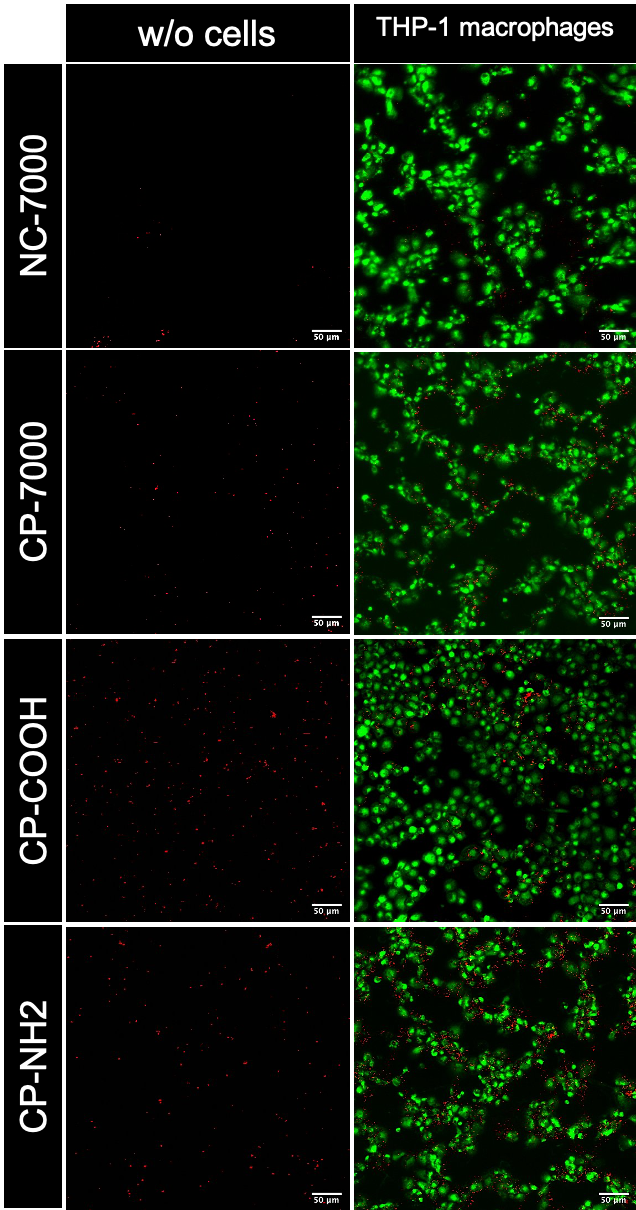
***

***Figure S3: FPLM detection of chemically purified MWCNT in absence or presence of macrophages.*** Femtosecond pulsed laser microscopy imaging of chemically purified MWCNT (red) deposited after 24 h in the absence (w/o, left) and presence (right) of THP-1 macrophages (green). Scale bars: 50 μm.

***
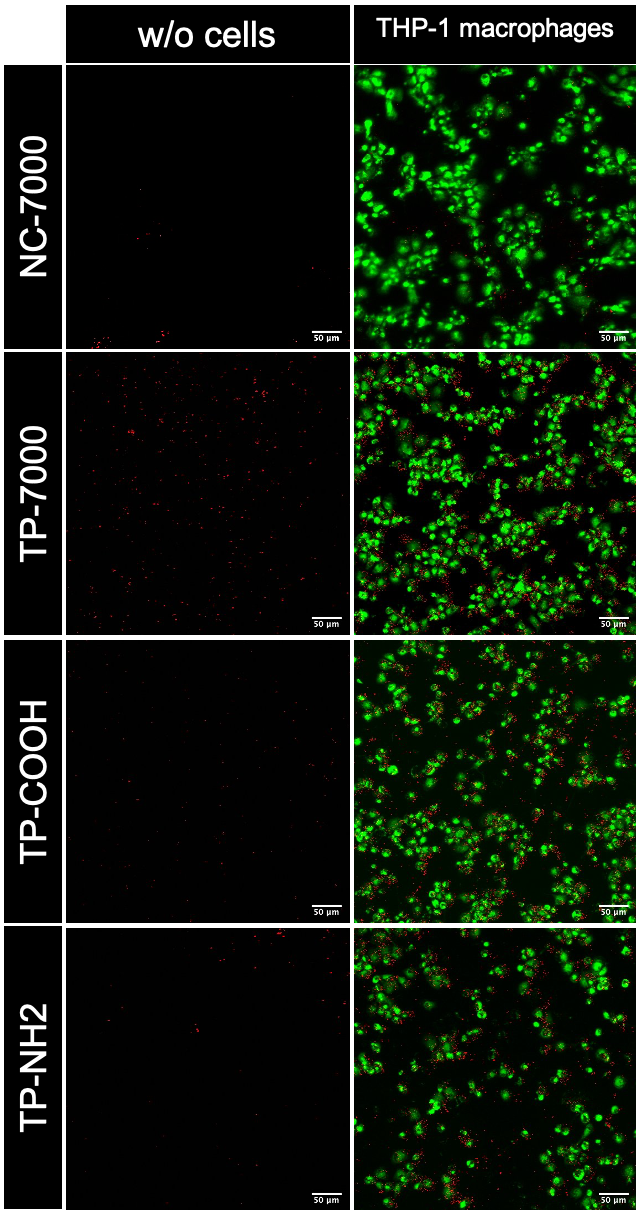
***

***Figure S4: FPLM detection of thermally purified MWCNT in absence or presence of macrophages.*** Femtosecond pulsed laser microscopy imaging of thermally purified MWCNT (red) deposited after 24 h in the absence (w/o, left) and presence (right) of THP-1 macrophages (green).

**
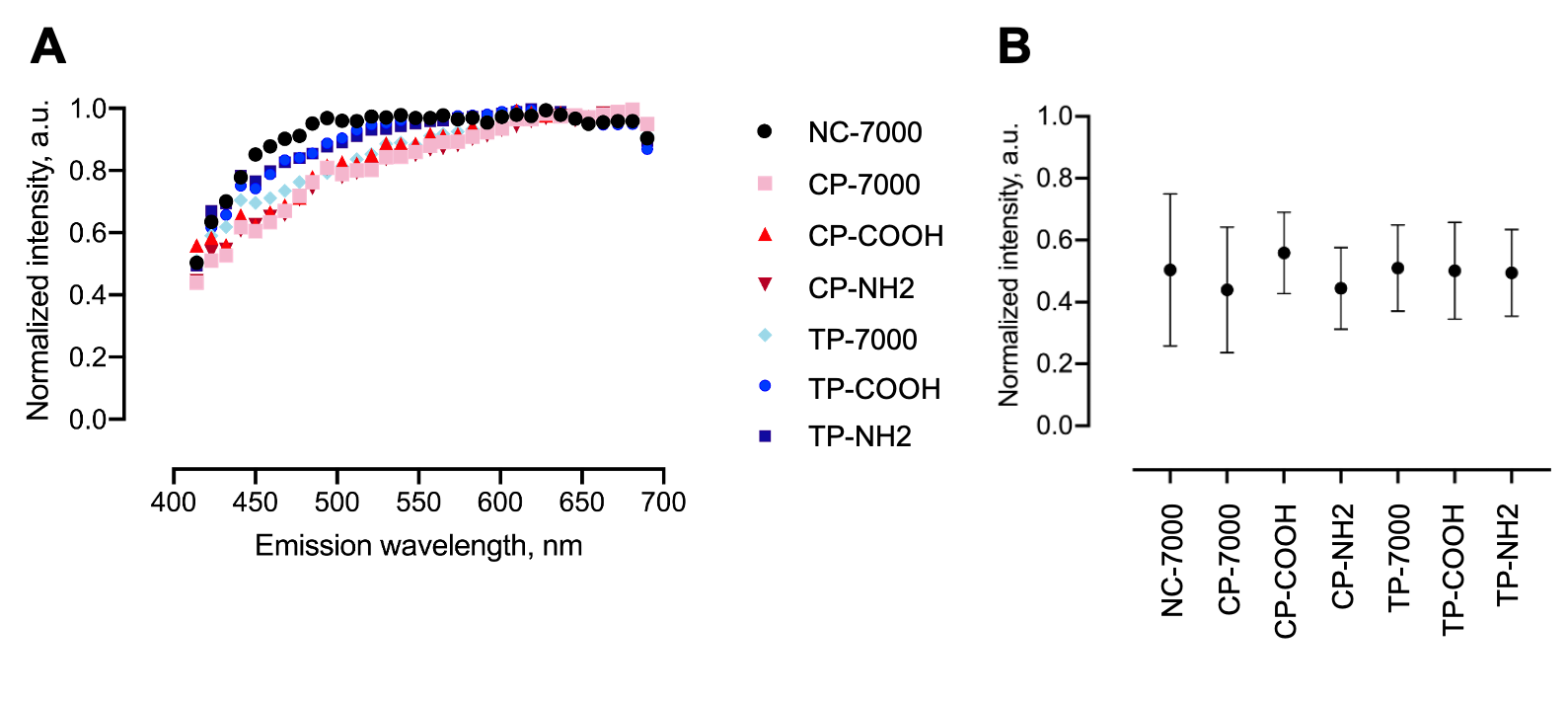
**

**Figure S5: Validation of the emission signal of the different MWCNTs.** (A) Emission fingerprint of the MWCNTs under femtosecond pulsed laser illumination (excitation 810 nm, 80 MHz, about 10 mW laser power on the sample) confirming the white-light generation of each MWCNT. Intensity normalized to the highest value of each spectrum. For each MWCNT, the spectrum represents the mean of 9 spectra (three technical replicates of three experimental replicates). Spectra are recorded from the wells without cells. (B) Comparison of the mean ± standard deviation intensity values of the spectrum recorded between 400 and 410 nm. No significant difference among the MWCNTs (one-way ANOVA followed by Tukey’s multiple comparison test).

1. Taylor-Just AJ, Ihrie MD, Duke KS, Lee HY, You DJ, Hussain S, et al. The pulmonary toxicity of carboxylated or aminated multi-walled carbon nanotubes in mice is determined by the prior purification method. Part Fibre Toxicol. 2020;17(1):60. [↑](#footnote-ref-1)
